# Adult Moyamoya Disease: A Burden of Intracranial Stenosis in East Asians?

**DOI:** 10.1371/journal.pone.0130663

**Published:** 2015-06-30

**Authors:** Oh Young Bang, Sookyung Ryoo, Suk Jae Kim, Chang Hyo Yoon, Jihoon Cha, Je Young Yeon, Keon Ha Kim, Gyeong-Moon Kim, Chin-Sang Chung, Kwang Ho Lee, Hyung Jin Shin, Chang-Seok Ki, Pyoung Jeon, Jong-Soo Kim, Seung Chyul Hong

**Affiliations:** 1 Department of Neurology, Samsung Medical Center, Sungkyunkwan University School of Medicine, Seoul, Korea; 2 Department of Radiology, Samsung Medical Center, Sungkyunkwan University School of Medicine, Seoul, Korea; 3 Department of Laboratory Medicine and Genetics, Samsung Medical Center, Sungkyunkwan University School of Medicine, Seoul, Korea; 4 Department of Neurosurgery, Samsung Medical Center, Sungkyunkwan University School of Medicine, Seoul, Korea; INSERM U894, FRANCE

## Abstract

**Background:**

Both Moyamoya disease (MMD) and intracranial atherosclerotic stenosis (ICAS) are more prevalent in Asians than in Westerners. We hypothesized that a substantial proportion of patients with adult-onset MMD were misclassified as having ICAS, which may in part explain the high prevalence of intracranial atherosclerotic stroke in Asians.

**Method:**

We analyzed 352 consecutive patients with ischemic events within the MCA distribution and relevant intracranial arterial stenosis, but no demonstrable carotid or cardiac embolism sources. Conventional angiography was performed in 249 (70.7%) patients, and the remains underwent MRA. The occurrence of the c.14429G>A (p.Arg4810Lys) variant in ring finger protein 213 (RNF213) was analyzed. This gene was recently identified as a susceptibility gene for MMD in East Asians.

**Results:**

The p.Arg4810Lys variant was observed in half of patients with intracranial stenosis (176 of 352, 50.0%), in no healthy control subjects (n = 51), and in 3.2% of stroke control subjects (4 of 124 patients with other etiologies). The presence of basal collaterals, bilateral involvement on angiography, and absence of diabetes were independently associated with the presence of the RNF213 variant. Among 131 patients who met all three diagnostic criteria and were diagnosed with MMD, three-fourths (75.6%) had this variant. However, a significant proportion of patients who met two criteria (57.7%), one criterion (28.6%), or no criteria (20.0%) also had this variant. Some of them developed typical angiographic findings of MMD on follow-up angiography.

**Conclusions:**

Careful consideration of MMD is needed when diagnosing ICAS because differential therapeutic strategies are required for these diseases and due to the limitations of the current diagnostic criteria for MMD.

## Introduction

Moyamoya disease (MMD) is a unique cerebrovascular disease characterized by progressive stenosis of the distal internal carotid artery (ICA) and a hazy network of basal collaterals called Moyamoya vessels. It was known that MMD mostly occurs in children in Asia, and the hemorrhage rate is higher among adults than children. However, recent epidemiologic studies of Asians and Westerners revealed that patients with MMD are older and more often ischemic or asymptomatic than previous studies indicated.[1] [2] [3] [4] One regional, all-inclusive data set of newly registered patients with MMD in Hokkaido (Japan, 2002 to 2006) showed that the percentage of patients less than 10 years of age at onset was 15% (compared to 48% in previous studies), and the highest peak was observed at 45–49 years. The data also revealed that the percentage of cases with ischemia increased to 57.4%. Only 21% (previously 42%) of adult MMD patients were hemorrhagic.[1]

Intracranial atherosclerotic stenosis (ICAS) is a common stroke subtype worldwide. Although ICAS is more prevalent in Asians than in Westerners, the reason for racial-ethnic differences is unknown. Possible explanations include inherited susceptibility to intracranial vessel atherosclerosis,[5] acquired differences in risk factor prevalence,[6] [7] and differential responses to the same risk factors.[8] [9] [10] Because both MMD and ICAS are more prevalent in Asians than in Westerners, the increased prevalence of ICAS may in part be caused by adult-onset MMD that is misclassified as ICAS.

We hypothesized that the current angiography-based criteria is limited in distinguishing MMD and ICAS, and that a substantial proportion of patients with adult-onset MMD are misclassified as having ICAS. A genome-wide linkage analysis and exome analysis recently identified ring finger protein 213 (*RNF213*) as the strongest susceptibility gene for MMD in East Asian people.[11] [12] Thus, we analyzed the occurrence of the p.Arg4810Lys variant of *RNF213* in relation to angiographic findings in adult stroke patients with intracranial arterial stenosis. In addition, the prevalence of this variant in non-stroke subjects and stroke patients was analyzed.

## Patients and Methods

### Patients

From January 2008 to November 2013, patients with ischemic cerebrovascular events in the middle cerebral artery (MCA) distribution who were admitted to the department of Neurology or Neursurgery at a University Medical Center were prospectively recruited. Potential participants were defined as patients experiencing focal or lateralizing symptoms within the MCA distribution and showing ≥50% stenosis or occlusion at terminal portions of the ICA and/or proximal anterior cerebral artery (ACA) and/or MCA on conventional or MR angiography. Based on the Stop Stroke Study Trial of Org 10172 in Acute Stroke Treatment (SSS-TOAST), patients with potential sources of cardioaortic embolism, extracranial atherosclerosis with significant (≥50%) stenosis on the relevant extracranial arteries, other stroke mechanisms (coagulopathy, vasculitis, arterial dissection, and others), or incomplete evaluations were excluded. Local institutional review boards approved this study. All patients or patient guardians provided informed consent for participation in this study.

### Workups

Clinical information including age, gender, and vascular risk factors was collected and all patients underwent diagnostic testing that included routine blood tests, electrocardiography, at least 24 hours of cardiac telemetry, and echocardiography. Vascular risk factors were defined as follows. 1) Hypertension was deemed present when the patient had been undergoing treatment with antihypertensive agents or their blood pressure was either ≥160 mm Hg systolic or ≤90 mm Hg diastolic on at least 2 occasions after the acute phase of their ischemic stroke. 2) Diabetes mellitus was deemed present when the patient had been receiving medication for diabetes, had an elevated fasting glucose level ≥126 mg/dL (7.0 mmol/L), a 2-hour plasma glucose ≥200 mg/dL (11.1 mmol/L) during an oral glucose tolerance test, or a plasma glucose level ≥200 mg/dL (11.1 mmol/L) along with the classic symptoms of hyperglycemia, hypoglycemic crisis, or hemoglobin A1c >6.5%. 3). Dyslipidemia was considered present if patients had been taking lipid-lowering agents or had a total cholesterol >240 mg/dL (6.21 mmol/L), triglycerides >200 mg/dL (2.26 mmol/L), or low-density lipoprotein cholesterol >160 mg/dL (4.14 mmol/L).

### Imaging analysis

A diagnosis of intracranial stenosis was made based on conventional angiography or magnetic resonance angiography (MRA). Conventional angiography was performed in 249 (70.7%) patients, especially when the presence of basal collaterals was highly suspicious or when vascular stenosis progression was observed on vascular studies. The remaining cases underwent MRA. Patients underwent comprehensive diagnostic cerebral angiography, including injection of internal and external carotid arteries and the dominant vertebral artery, with image acquisition in the late venous phase to assess collateral circulation from all possible sources. Three-dimensional time-of-flight MRA of the intracranial arteries (repetition time 25 msec, echo time 3.5 msec, 80 slices of 0.45-mm thickness over contiguous sampling, 20° flip angle, a 880 ᵡ 450 matrix, and field of view 170 mm) and gadolinium-enhanced MRA of the extracranial arteries were obtained for all patients (3-T, Achieva; Philips Medical System, Best, the Netherlands). Diagnosis of MMD was based on the characteristic angiographic appearance of stenosis and basal collaterals (Table 1). [12]

**Table 1:** Diagnostic criteria and their limitations in adult MMD.

Diagnostic criteria	Limitations in adults
1. Steno-occlusive lesions around terminal portions of the ICA.	Stenosis of the distal intracranial ICA and tandem stenosis of the proximal ACA and MCA may not be observed in the early stages of MMD (Fig 2).
2. Moyamoya vessels at the base of the brain appearing as abnormal vascular networks on conventional angiography or MR angiography.	No objective criteria for ‘prominent’ basal collateral vessels.
	Less prominent basal collaterals in adult MMD than childhood MMD
3. Findings 1 and 2 are present bilaterally (definite MMD according to diagnostic criteria).	Contralateral disease develops in up to 40% of patients with unilateral MMD.[33] [34] [35] (Fig 2)
4. Exclusion of known disease with similar angiographic findings (arteriosclerosis, autoimmune disease, meningitis, brain neoplasm, Down syndrome, neurofibromatosis type 1, head trauma, head irradiation, and protein C or S deficiency).	Relatively common steno-occlusive diseases causing ‘Moyamoya syndrome’ (e.g., intracranial atherosclerosis) in adults.

### Identification of *RNF213* Mutations

Genomic DNA was extracted from peripheral blood leukocytes using a Wizard Genomic DNA Purification kit and following the manufacturer’s instructions (Promega, Madison, WI). The c.14429G>A (p.Arg4810Lys) mutation of the *RNF213* gene (Genebank accession number NM_001256071.1) was amplified using primer sets designed by the authors (available upon request). Polymerase chain reaction (PCR) was performed with a thermal cycler (model 9700, Applied Biosystems, Foster City, CA, USA), and direct sequencing was performed with a BigDye Terminator Cycle Sequencing Ready Reaction kit (Applied Biosystems) on an ABI Prism 3730*xl* genetic analyzer (Applied Biosystems).

### Statistics

Commercially available software (SPSS, version 18.0; SPSS Inc., Chicago, IL) was used for statistical analyses. Differences in discrete variables among the groups were examined via χ^2^, Fisher exact, and Mann-Whitney tests. Differences in continuous variables were examined using 1-way analyses of variance, Kruskal-Wallis tests, and *t* tests. In addition, independent factors for *RNF213* mutation were evaluated using logistic regression. Univariate analyses variables with *P<*0.2 were considered explanatory variables and were evaluated together in subsequent multivariate analyses. *P<*0.05 was considered statistically significant.

## Results

### General characteristics

Of 352 patients with intracranial stenosis, 220 (62.5%) were female and the average age was 51.3 ± 13.7 (ranging from 22- to 93-years-old). One hundred thirty-one patients were diagnosed with MMD, whereas 221 had ICAS. Steno-occlusive lesions on the terminal ICA were observed in 220 patients, while 107 patients had proximal MCA lesions, 5 had proximal ACA lesions, and 21 had both MCA and ACA lesions.

### *RNF213* variant

The p.Arg4810Lys variant was observed in 176 (50.0%) patients with intracranial stenosis (175 heterozygotes and 1 homozygote), in no healthy control patients (n = 51) and in 3.2% of stroke control patients (4 heterozygote of 124 patients). The latter included lacunar stroke (n = 44), cardioembolism (n = 46), cervical carotid atherosclerosis (n = 8), and other etiologies (n = 25).

Table 2 shows patient characteristics depending on the presence of the *RNF213* variant. Patients with the *RNF213* variant were younger, while female gender and diabetes was more prevalent in patients without this variant. No differences in other risk profiles were found among the groups. All angiographic diagnostic criteria, including involvement of distal ICA, presence of basal collaterals, and bilateral involvement, were more frequently observed in patients with the *RNF213* variant. Multivariate testing was performed to further evaluate independent predictors for the presence of the *RNF213* variant. The presence of basal collaterals, bilateral involvement on angiography, and absence of diabetes were independently associated with the presence of the *RNF213* variant.

**Table 2:** Factors predicting the genetic variant of *RNF213* associated with MMD among 352 patients with intracranial arterial stenosis.

	*RNF 213*			Univariate		Multivariate	
	present	absent	P-value	OR (95% CI)	P-value	OR (95% CI)	P-value
Age	46.5 ± 12.2	57.4 ± 14.9	<0.001	0.977 (0.956–0.999)	0.040	0.980 (0.960–1.000)	0.052
Female gender	121 (67.2%)	149 (50.3%)	<0.001	1.148 (0.677–1.948)	0.607		
Family history of MMD	14 (7.8%)	4 (1.4%)	<0.001	2.905 (0.831–10.159)	0.095	2.828 (0.824–9.707)	0.099
Vascular risk factors							
Hypertension	85 (47.2%)	161 (54.4%)	0.129	1.226 (0.713–2.108)	0.460		
Diabetes	21 (11.7%)	78 (26.4%)	<0.001	0.349 (0.178–0.684)	0.002	0.368 (0.192–0.704)	0.003
Dyslipidemia	51 (28.3%)	96 (32.4%)	0348	1.025 (0.585–1.795)	0.931		
Angiographic findings							
Distal ICA involvement	138 (76.7%)	91 (30.7%)	<0.001	1.569 (0.866–2.842)	0.137		
Basal collaterals	134 (74.4%)	57 (19.3%)	<0.001	3.027 (1.714–5.346)	<0.001	3.529 (2.066–6.026)	<0.001
Bilateral involvement	128 (71.1%)	65 (22.0%)	<0.001	2.625 (1.510–4.564)	0.001	3.072 (1.825–5.170)	<0.001
No. diagnostic criteria met*			<0.001				
0	16 (20.0%)	64 (80.0%)		Ref		Ref	
1	20 (28.6%)	50 (71.4%)		1.542 (0.701–3.388)	0.281	1.529 (0.699–3.348)	0.228
2	41 (57.7%)	30 (42.3%)		4.834 (2.244–10.412)	<0.001	4.797 (2.236–10.289)	<0.001
All 3	99 (75.6%)	32 (24.4%)		9.753 (4.687–20.294)	<0.001	10.214 (4.952–21.068)	<0.001

* Model 2, including age, gender, family history of MMD, vascular risk factors, and number of angiographic diagnostic criteria met.

**Fig 1:**
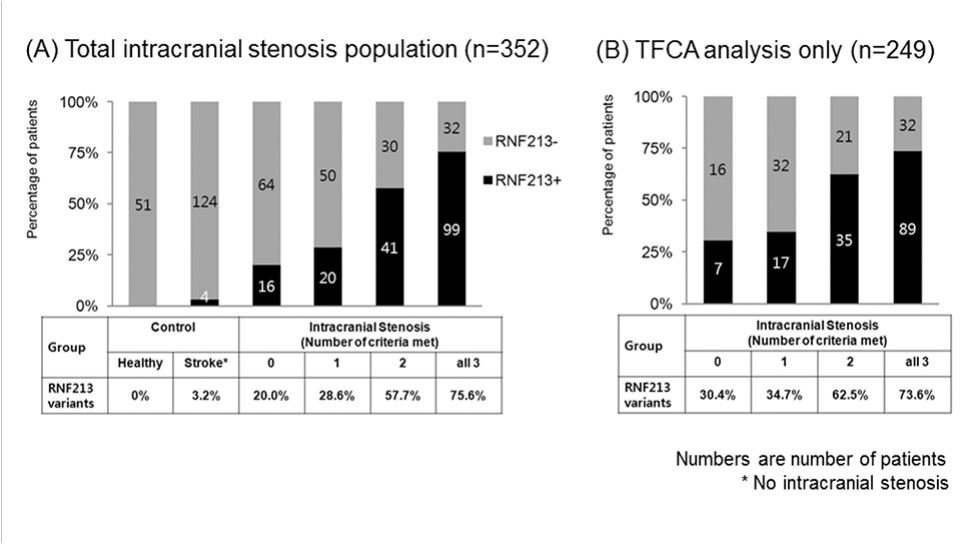
Number of cases with *RNF213* variant+ among intracranial patients with healthy and stroke controls.

**Fig 2:**
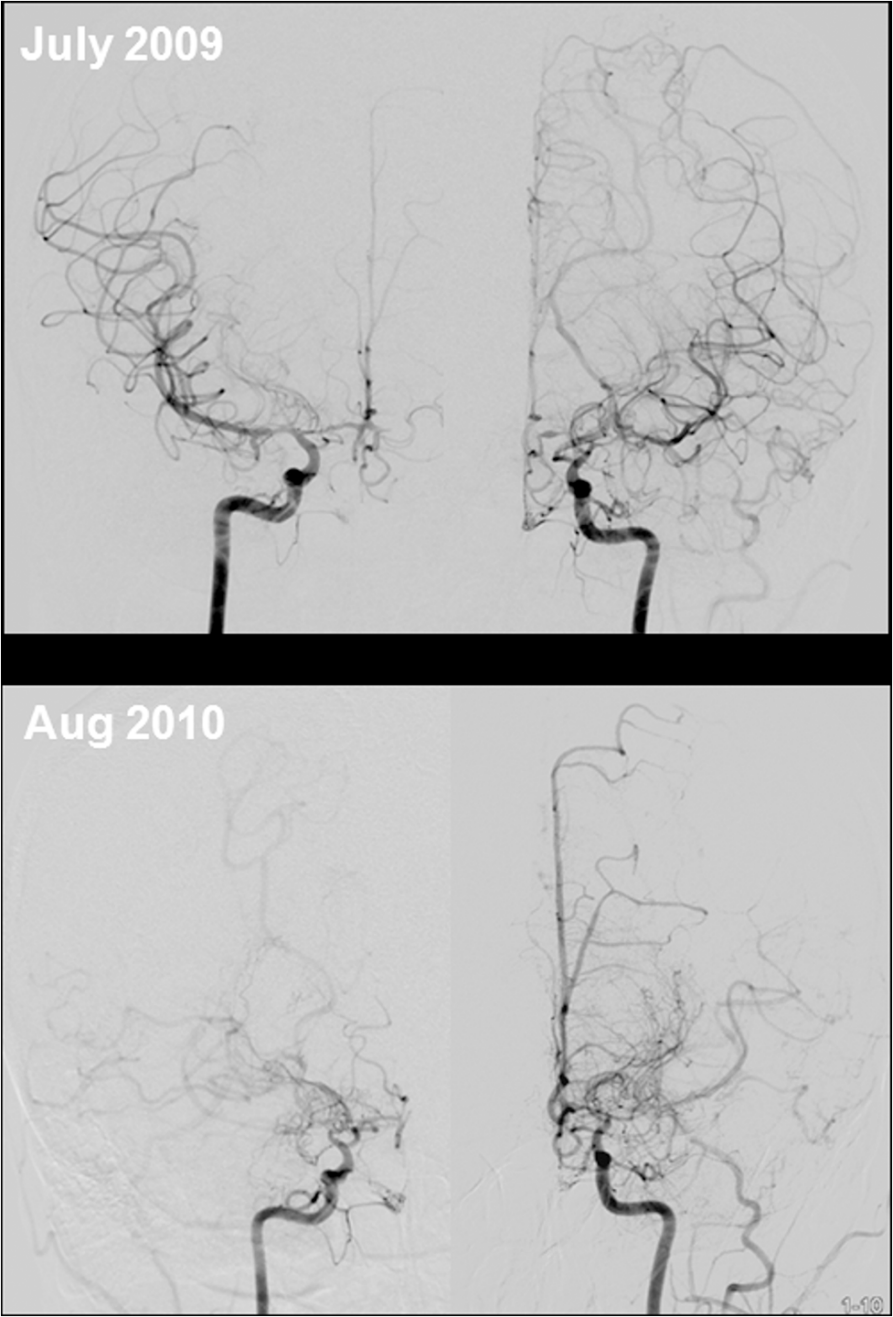
Angiographic progression of MMD in an adult patient with intracranial stenosis. A 42-year-old female presented with transient numbness and clumsiness of her left hand. She had mild stenosis on bilateral and proximal middle cerebral arteries. There was no stenosis of the distal internal carotid artery and basal collaterals, called Moyamoya vessels, on conventional angiography (upper lane). Angiographic findings taken one year later show the progression of stenosis and Moyamoya vessels bilaterally (lower lane). Genetic study revealed *RNF213* mutation associated with MMD (p.Arg4810Lys).

**Fig 3:**
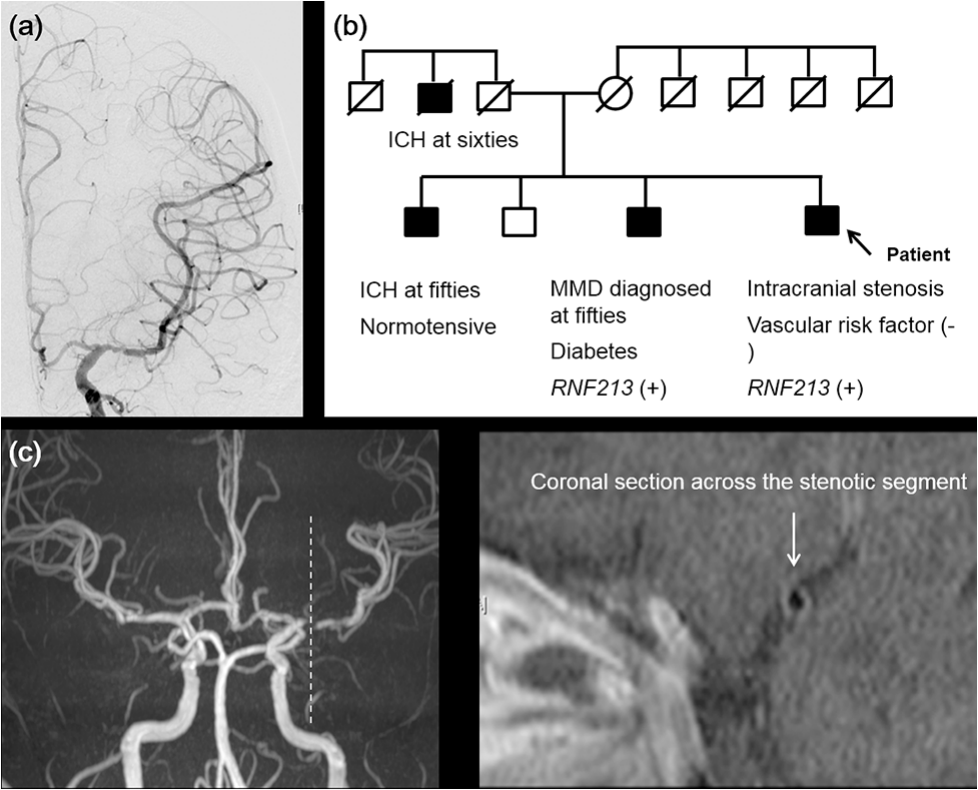
Neuroimaging and genetic findings of a patient with non-Moyamoya-type intracranial arterial occlusive disease. (a) Conventional angiography of a 53-year-old male shows stenosis of the proximal MCA, but intact distal ICA and absence of Moyamoya vessels. (b) Family tree. This patient has a family history of MMD and *RNF213* p.Arg4810Lys mutations. Small black points indicate members who were directly examined. (c) High-resolution MRI reveals a smaller outer diameter (2.32 mm) and the absence of focal plaque in the stenotic segment (arrow). ICH, intracranial hemorrhage.

Among 131 patients who met all three diagnostic criteria and were diagnosed with MMD, three fourths (75.6%) had the *RNF213* variant. The *RNF213* variant was also observed in a significant proportion of patients who did not meet the diagnostic criteria of MMD and met two (57.7%), one (28.6%), or none (20.0%) of the angiographic criteria (Table 2 and Fig 1A). Such findings were consistently observed among patients who underwent cerebral angiography (Fig 1B). Some patients developed typical angiographic MMD findings, such as basal collaterals on follow up angiography (Fig 2). One patient who met only one criterion, but had a family history of MMD underwent high-resolution MRI, which showed shrinkage and absence of atherosclerotic plaque in the stenotic MCA segment (Fig 3).

## Discussion

The major findings of this study are (a) there is a prevalent genetic variant associated with MMD, constituting half of patients with intracranial arterial stenosis, (b) there is no clear cutoff point for angiographic-based diagnostic criteria of MMD; this genetic variant was observed in 3/4 of patients with all three criteria (definite MMD), but also in 1/2 of patients with two criteria, and in 1/4 of patients with one criterion who were diagnosed with ICAS, (c) some patients may show characteristic features of MMD on high-resolution MRI or serial angiographic studies.

Risk factor control, aggressive medical management (including statins), and stent placement (in selected patients) are important for preventing stroke in patients with ICAS.[13] [14] The pathophysiology of MMD is still unknown, and no medication can stop or reverse its progression. Several case series consistently showed that the role of stenting in MMD is highly questionable and is associated with a high rate of symptomatic restenosis/occlusion.[15] [16] [17] [18] Revascularization surgery remains the mainstay of treatment for MMD, whereas the recent guidelines do not recommend bypass surgery for ICAS.[19] Therefore, differentiation of MMD from ICAS is important for treating patients with intracranial occlusive disease.

However, it is often difficult to differentiate these conditions in adult patients with intracranial arterial stenosis. There is a paucity of data on adult MMD, and most is based on childhood MMD data, including angiographic criteria for diagnosis. The criteria for diagnosing MMD set forth in 1997[20] may have limitations (Table 1). First, in the current diagnostic criteria, prominent basal collaterals are required for diagnosis. However, the presence of basal collaterals is subjectively determined because there is no established definition of ‘prominent’ basal collateral vessels. Unlike to childhood-onset MMD, the length of basal collaterals often less prominent in adult-onset MMD suggest that the degree of angiogenesis is different depending on age at onset. Angiogenesis (development of Moyamoya vessels) represents either a compensatory mechanism for reduced cerebral blood flow or aberrant active angiogenesis before vascular occlusion. Angiogenesis and collateral vessel development are reportedly impaired with aging, which may be due to age-related endothelial dysfunction and reduced angiogenic cytokine expression.[21] Second, patients may present with unilateral MMD or have stenotic lesions in the MCA or ACA with a relatively intact distal ICA. In patients with MMD, angiographic findings differ according to the progressive stage, and characteristic angiographic findings are not consistently observed in all courses of MMD (Fig 2).[20, 22] Moreover, the progression rate may differ for childhood and adult-onset MMD. Contralateral progression tended to occur within 3 years of initial diagnosis in children aged <9 years.[23] Conversely, the rate and time of contralateral progression in adult MMD is unsettled. Lastly, adult patients with MMD often have vascular risk factors, unlike patients with childhood MMD.

The *RNF213* genetic variant was identified in 95% of patients with familial MMD, 80% with sporadic MMD, and 1.8% of control patients.[24] The stroke control and healthy control subjects showed similar results. Recently, Miyawaki and colleagues suggested that a particular subset of Japanese patients with non-MMD intracranial stenosis has a genetic variant associated with MMD.[25] [26] In their studies, 22–24% of ICAS patients have the *RNF213* genetic variant associated with MMD. However, these patients may have MMD rather than ICAS even though they did not have signs of MMD (e.g., basal collaterals). The present study showed that there is no clear cutoff point for angiographic-based diagnostic criteria of MMD. The occurrence of this genetic variant increased with increasing amounts of observed angiographic criteria, and 43% (61 of 141) of patients who had only one or two of three characteristic angiographic findings and were diagnosed with ICAS had this genetic variant. Our presenting cases showed that patients may have typical angiographic findings during the follow-up period, and high-resolution MRI may reveal characteristic vessel wall changes of MMD (shrinkage) rather than atherosclerotic plaque on the stenotic segment.[27] [28]

Results of the present study raise the possibility that misclassification of adult-onset MMD as ICAS could contribute to the high prevalence of intracranial atherosclerotic stroke in Asians. The population susceptible to MMD was estimated as 16.16 million people in East Asian countries.[29] The number of patients with MMD, which was estimated conservatively at 1 per 300 carriers of the *RNF213* p.Arg4810Lys variant, was considered to be 53,800 people in East Asian populations.[30] [29]

This study has several limitations. First, although all patients underwent comprehensive workups, including vascular, laboratory, and cardiologic evaluations, the study was cross-sectional and had a limited sample size. Serial vascular studies were performed in selected patients, including transcranial Doppler and magnetic resonance angiography. Second, conventional angiography was not performed in all patients. However, more than two-thirds of patients with intracranial stenosis underwent conventional angiography. Axial source images of time-of-flight MR angiography were analyzed to evaluate moyamoya vessels at the level of the Sylvian valley in patients who underwent MRA alone. Third, participants in our study are not representative of the general population with intracranial stenosis because this is a single center study at a tertiary referral center where bypass surgery for MMD is actively preformed. Moreover, the results of this study cannot be generalized outside of East Asians because *RNF213* p.Arg4810Lys variant is not the susceptibility gene for MMD in Westerners or South Asians. In addition, clinical manifestations and angiographic findings may differ for Westerners and East Asians.[31] Last but not the least, MMD may be a multifactorial phenomenon and there may be many different causes of the underling occlusive vasculopathy. The *RNF213* genetic variant could lead to vascular fragility (including medial thinness), which may make vessels more vulnerable to hemodynamic stress and secondary insults, and these genetic variant also facilitate the development of the formation of basal collaterals in the setting of large intracranial arterial stenosis, including ICAS.[32]

In conclusion, our data indicate that a more careful consideration of MMD is needed when diagnosing ICAS because the therapeutic strategies between these disease processes differ and the current diagnostic criteria for MMD are limited. In adult patients with intracranial occlusive disease, diagnostic criteria based on molecular or mechanistic classification rather than angiographic findings may be needed. We have an ongoing prospective follow-up study of patients with apparent ICAS involving multimodal biomarkers (NCT02074111 at Clinical.trial@gov). Variants in RNF213 in non-p.Arg4810Lys were recently found in Caucasian and Chinese cases with MMD.[12] Further genetic studies for MMD are warranted in populations outside East Asia.

## Supporting Information

S1 File BOY Supplementary. (PDF)
