# Supplementary material for: Adult Moyamoya Disease: A Burden of Intracranial Stenosis in East Asians?
Source: PLoS One. 2015 Jun 30;10(6):e0130663. doi: 10.1371/journal.pone.0130663 (PMC4488323; doi:10.1371/journal.pone.0130663)

SUPPLEMENTARY MATERIAL

Supplementary figure. Neuroimaging and genetic findings of a patient with non-Moyamoya-type intracranial arterial occlusive disease.

(a) Conventional angiography of a 53-year-old male shows stenosis of the proximal MCA, but intact distal ICA and absence of Moyamoya vessels. (b) Family tree. This patient has a family history of MMD and *RNF213* p.Arg4810Lys mutations. Small black points indicate members who were directly examined. (c) High-resolution MRI reveals a smaller outer diameter (2.32 mm) and the absence of focal plaque in the stenotic segment (arrow). ICH, intracranial hemorrhage.

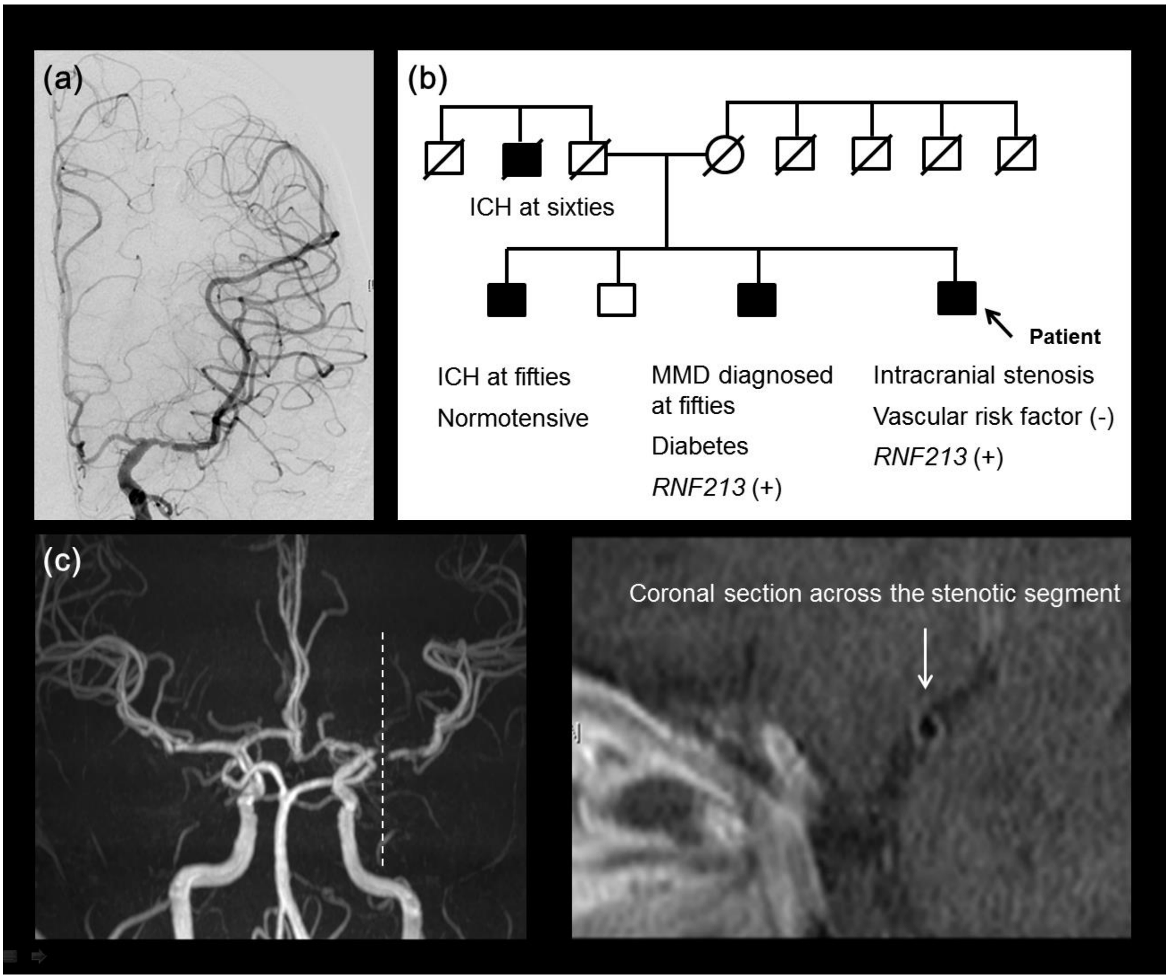

Supplement: S1 File — (PDF) [file pone.0130663.s001.pdf]
